# 1‐Methylcyclopropene (1‐MCP) retards the senescence of *Pteridium aquilinum var. latiusculum* by regulating the cellular energy status and membrane lipid metabolism

**DOI:** 10.1002/fsn3.2406

**Published:** 2021-06-23

**Authors:** Wentao Zhang, Zhen Li, Meiling Du, Xiuling Zhang, Yaqin Tian, Jinge Wang

**Affiliations:** ^1^ College of Food Science Northeast Agricultural University Harbin PR China

**Keywords:** 1‐MCP, energy metabolism, membrane fatty acids, membrane lipid metabolism, *Pteridium aquilinum var. latiusculum*

## Abstract

1‐MCP is an ethylene inhibitor which can delay the ripening and senescence of fruits and vegetables effectively. *Pteridium aquilinum var. Latiusculum* (PA) is one of the wild vegetables which is famous and nutrient in China. However, the mechanism of PA preservation treated with 1‐MCP has not been reported. Consequently, the effects of postharvest 1‐MCP treatment on the changes in quality, energy metabolism, and membrane lipid metabolism of PA were investigated in this study. The results indicated that 1‐MCP treatment could effectively inhibit the decreases in firmness, titratable acid (TA) content and the increases in weight loss rate, malondialdehyde (MDA) content, membrane permeability, and membrane lipid metabolism‐related enzymes in PA. The cellular energy charge (EC) and the levels of ATP, ATP/ADP, and ATP/AMP, the activities of energy metabolism‐related enzymes, NAD^+^, and NADH were maintained, and the decreases in unsaturated fatty acids and the ratio of unsaturated‐to‐saturated fatty acids in the membrane of PA cells were effectively retarded by 1‐MCP treatment. A positive correlation was observed between cellular ATP levels and the ratio of unsaturated‐to‐saturated fatty acids, while negative correlations were observed between the ratio of unsaturated‐to‐saturated fatty acids and both lipid peroxidation and membrane permeability. These results indicated that higher levels of energy status, unsaturated‐to‐saturated fatty acid ratios, and lipid metabolism in the membrane could preserve the membrane integrity of postharvest PA and effectively extend its shelf life.

## Graphical abstract

1‐MCP treatment could affect the quality, energy status, membrane lipid metabolism, membrane fatty acids, the activities of energy‐metabolism‐related enzymes, and lipid metabolism‐related enzymes of postharvest *Pteridium aquilinum var. latiusculum* (PA). The results indicated that 1‐MCP treatment could extend the shelf life of PA effectively.

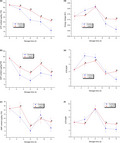

## 1 INTRODUCTION

*Pteridium aquilinum var. latiusculum* (PA) is a popular fern species in the Athyriaceae family that is widely distributed in Northeast China. It is rich in nutrients and exhibits the abilities to promote the growth and development of children, improve body immunity, strengthen memory, and delay the aging of the brain (Yamada et al., 2007). Because of its classification as a natural food and its abundance of nutrients, it has attracted increasing numbers of consumers in recent years. In 2015, the consumption of wild vegetables in Heilongjiang Province of China reached approximately forty‐one thousand tons. Although the selling price of PA is increasing year by year, it is still popular in both domestic and international markets. It has become the leading variety of wild vegetables for export in Northeast China, creating great economic value (Hao et al., 2021). Nevertheless, PA usually grows deep in the mountains and woodlands that are far from the sales places. Ripening and senescence during transport cause considerable losses not only for PA but also for other wild vegetables.

Fruits and vegetables are still living organisms carrying out a series of living activities after harvest. Ripening and senescence processes easily occur due to the transient harvest period of PA during spring in Northeast China, which creates difficulties for its storage and preservation. Previous studies indicated that the energy supply of fruit and vegetable cells affects a series of physiological metabolic possesses during postharvest ripening and that postharvest quality changes were closely related to the energy dissipation of fruit and vegetable cells (Aghdam et al., 2018; Jin et al., 2014; Qu et al., 2006; Saquet et al., 2000; Wang et al., 2013). Adenosine triphosphate (ATP) is the primary energy resource found in cells. It plays an important role in maintaining the membrane integrity and energy status change of postharvest fruit and vegetable cells during the ripening process (Saquet et al., 2000, 2003). Some previous studies have also found that membrane integrity affects the browning of fresh‐cut pears and litchi fruit (Jiang et al., 2018; Li et al., 2017). Therefore, changes in the energy metabolism within cells could affect the quality and decay of postharvest crops by impacting the integrity of cell membranes.

1‐Methylcyclopropene (1‐MCP) is an effective preservative for fruits and vegetables since the ripening and senescence of fruits and vegetables will be inhibited. 1‐MCP has the advantages of high efficiency, safety, nontoxicity, and no residue. It is currently widely used in postharvest horticultural products. There have been sufficient studies to show that 1‐MCP treatment can extend shelf life efficiently (Kou et al., 2020; Kubo et al., 2003; Luo, 2007; Mata et al., 2018; Rocculi et al., 2009; Sharma et al., 2010; Sivakumar et al., 2010; Zhu et al., 2013).

Although 1‐MCP has been widely used to improve the quality of postharvest vegetables, no study has focused on changes in the energy status, membrane fatty acid content, and membrane lipid metabolism of PA after 1‐MCP treatment. Therefore, the objective of this study was to explore the relationship between the changes in energy status, membrane fatty acids, membrane lipid metabolism, and quality ripening of PA after 1‐MCP treatment.

## 2 MATERIALS AND METHODS

### 2.1 Experimental materials and treatments

All the PA materials for the experiment were harvested from Heilongjiang Beian Agricultural Reclamation Tianyunshan Products Co., Ltd of Heihe, within Heilongjiang Province, China. All the PA materials were harvested on May 15th, 2020. The collection was based on their uniformity of size and maturity with the absence of diseases and mechanical damage. The length of each PA was between 20 and 25 cm. The whole harvesting process followed the local standards of Heilongjiang Province (Technical regulation for harvesting and postharvest treatment of *Pteridium aquilinum var. latiusculum*). The samples were quickly transported to the College of Food Science, Northeast Agricultural University after harvesting.

PA samples were rinsed using distilled water to remove dirt and dust on the surface. After air drying, 400 samples were evenly divided into the treatment group and control group. All the samples in the treatment group were fumigated using a 1 μL/L 1‐MCP solution for 24 hr in an incubator (RGX‐80B, Shanghai Kuntian Laboratory Instrument Co., Ltd) without controlled atmosphere according to the results of our previous experiment. Others in the control group were fumigated by distilled water. After fumigation, all the samples in both groups were transferred to 4℃ and 90%–95% relative humidity (RH) conditions. High RH is arranged to reduce the effect on energy level and membrane integrity of water loss. All the determinations were performed every three days, and each treatment contained three biological repeats to guarantee the accuracy and reliability of the determination.

### 2.2 Determination of firmness, titratable acidity, weight loss rate, and ethylene production

The PA firmness was measured using a texture analyzer with a probe with a diameter of 2 mm (TA‐XTplus2, Stable Micro Systems Ltd, UK). The puncture speed and puncture distance of the texture analyzer were 2 mm/s and 12 mm, respectively. Ten samples were selected randomly from each treatment to calculate their average. Each sample was straightened before determination. The firmness was measured at the middle point of each PA sample length. Firmness was expressed as N.

Randomly selected PA tissue (10 g) from each group was used to measure the titratable acid (TA) content of PA. Samples were put into a mortar to be homogenized by continuous grinding. Then, all the residue was transferred to a 100 ml volumetric flask and filtered 30 min later. A mixture of 20 ml filtrate and two drops of 1% phenolphthalein indicator was titrated by 0.1 M sodium hydroxide. Titration was not considered complete until the solution appeared pink and did not fade within 0.5 min (pH=8.1–8.3). The amount of sodium hydroxide solution was recorded, and this step was repeated three times to guarantee accuracy. Finally, titration of distilled water instead of filtrate was used as a blank control. The TA content was calculated by the following equation:

$$\text{Total TA content}=\frac{V\times c\times (V_{1}‐V_{0})}{Vs\times m}$$

*V* represents the total volume of the sample extraction solution (mL); *Vs* represents the filtrate volume (mL); *c* represents the concentration of sodium hydroxide solution (mol/L); *V*_1_ represents the volume of sodium hydroxide solution needed to titrate the filtrate (mL); *V*_0_ represents the volume of sodium hydroxide solution needed to titrate distilled water (mL); and *m* represents the sample quality (g).

The weight loss rate of PA in both two groups was calculated according to the following equation:

$$\text{Weight loss rate}=\frac{m_{0}‐m_{i}}{m_{0}}$$

*m*_0_ represents the original weight of PA sample before the storage; *m_i_* represents the weight of each determination during the storage.

The determination of ethylene production was measured as following steps: 15–20 PAs were randomly chosen in each group and placed into sealed glasses, respectively. 1 ml gas from the head‐space of the container was taken and measured using a gas chromatographer (Agilent 7890B). The temperatures of the oven, gasifier, and FID were at 90℃, 140℃, and 200℃, respectively. Carrier flow rates of nitrogen, hydrogen, and air were 30, 30, and 300 ml/min, respectively.

### 2.3 Determination of lipid peroxidation and membrane permeability

Malondialdehyde (MDA) is one of the main products generated from membrane lipid peroxidation. The MDA content is usually regarded as an index that reflects the extent of membrane lipid peroxidation. The reaction of MDA with proteins and nucleic acids changed the configuration of these macromolecules and resulted in a loss of their biological functions. Therefore, the accumulation of MDA could cause damage to the membrane and organelles to a certain extent.

A mixture of 1.0 g of PA sample tissue and 5.0 ml of 100 g/L trichloroacetic acid (TCA) solution was ground. The supernatant was extracted from the homogenate by centrifugation at 4℃ and 10,000 × g for 20 min. Then, 2.0 ml of 0.67% thiobarbituric acid (TBA) was added to 2.0 ml of supernatant. After mixing, the mixture was boiled in a boiling water bath for 20 min. Then, it was quickly cooled in an ice bath and centrifuged one more time (4℃, 10,000 × g for 20 min). The absorbance of the supernatant was recorded at 450, 532, and 600 nm. The MDA concentration of the sample extract could be calculated by means of the following equation:

$$c\left(\mu \text{mol}/L\right)=6.45\times \left({\text{OD}}_{532}‐{\text{OD}}_{600}\right)‐0.56\times {\text{OD}}_{450}$$

Then, the MDA content of the sample extract could be calculated according to the following equation. The results were expressed as μmol/g on a fresh weight (FW) basis:

$$\text{MDA content}=\frac{c\times V}{Vs\times m\times 1000}\left(\text{mmol}/\text{kg}\right)$$

*c* represents the MDA content of the reaction mixture (mmol/kg); *V* represents the total volume of the sample extract (mL); *Vs* represents the total volume of the sample extract required for determination (mL); and *m* represents the sample weight (g).

The membrane permeability of PA was measured using the relative leakage rate (RLR). The sample tissue disks were removed using a hole punch from the equatorial region of each PA. Ten disks from each of the samples from each treatment were immersed in 20 ml of deionized water at room temperature for 30 min and then vibrated for 10 min. The samples were removed, and the remaining water on the surface was dried using filter paper. Then, the samples were put into a boiling tube and immersed in 20 ml of deionized water again. The initial electrical conductivity of the solution was measured using a conductivity meter at a constant temperature of 20℃–25℃, and it was represented by *γ*_1_. Then, the solution with disks was moved into a boiling water bath for 15 min and allowed to cool, and the final electrical conductivity of the solution was measured at a constant temperature of 20℃–25℃. The final electrical conductivity of the solution was represented by *γ*_0_. The relative electrical conductivity was represented by *γ*_e_ and was calculated by the following equation:

$$\gamma_{e}=\frac{\gamma 1}{\gamma 0}\times 100\%$$

### 2.4 HPLC analysis of ATP, ADP, and AMP

The extraction and determination of ATP, ADP, and AMP were executed according to the method of Huang et al., (2019) with slight modifications. First, 4 ml of 0.4 M perchloric acid was added to 5.0 g of homogeneous PA sample tissues and then centrifuged at 4℃ and 10,000 × g for 15 min. 3 ml of supernatant was quickly neutralized to pH 6.5 using 1 M potassium hydroxide, diluted to 15 ml, and then filtered through a 0.45 μm filter membrane. The filtrate was transferred and stored at −30℃ until determination.

The determination of ATP, ADP, and AMP was executed using an HPLC system (Surwit) with an RPL‐D2000 C18 column (0.5 μm × 4.6 mm × 250 mm). Mobile phase A contained 0.06 M dipotassium hydrogen phosphate and 0.04 M potassium dihydrogen phosphate dissolved in deionized water and adjusted to pH 7.0 with 0.1 M hydrochloric acid. Mobile phase B was pure acetonitrile. Elution was executed using a linear gradient program with 75% to 100% A and 0% to 25% B for 7 min. The temperature of the column oven was 25℃. The flow rate was 1.0 ml/min. 15 μL of each sample was injected into the HPLC system for the analysis of ATP, ADP, and AMP concentrations according to the external standard curve. The energy charge was calculated by the following equation:

$$\text{Energy charge}\;=\frac{\text{ATP}+0.5\times \text{ADP}}{\text{ATP}+\text{ADP}+\text{AMP}}$$

The results were expressed in μg/kg.

### 2.5 Determination and analysis of membrane fatty acids

The membrane lipids were extracted and quantified according to Huang et al., (2019) with slight modification. 2.0 g of homogenized PA sample tissues was transferred to a flask and mixed with 2.0 ml of internal standard solution (triglyceride undecarbonate). Then, 100 mg of pyrogallic acid, 2 ml of 95% ethanol, and 4 ml of distilled water were added to the solution sequentially. 8 ml of a 2% NaOH–methanol solution was added to the extraction and then connected to a reflux condenser. Reflux was performed in an 80℃ water bath until the oil disappeared. 7 ml of 15% boron trifluoride–methanol solution was added from the top of the reflux condenser and refluxed in an 80℃ water bath for 2 min. Then, the flask was removed from the water bath and cooled to room temperature immediately. Next, 10–30 ml of n‐heptane was added to the solution and vibrated for 2 min. After vibration, a saturated NaCl solution was added, and the solution was separated into different layers. 5 ml of from the top of the n‐heptane extraction solution was transferred to a test tube, and then 5 g of Na_2_SO_4_ was added with vibration for 1 min. After standing for 5 min, the solution in the upper layer was used to test the content of fatty acids.

Fatty acids were separated and quantified using gas chromatography (Agilent, 7890B GC system) equipped with a flame ionization detector (FID) and an SP‐2560 column. The specifications of the SP‐2560 column were 100 m × 250 μm (internal diameter) × 0.2 μm film thickness. The temperature program was set as follows: the initial temperature of 140℃ was held for 5 min; the sample was heated at a rate of 10℃ min^−1^ from 140℃ to 180℃ and held for 6 min when the temperature reached 180℃; the sample was then heated from 180℃ to 200℃ at a heating rate of 2℃ min^−1^ and held for 20 min when the temperature reached 200℃; finally, the sample was heated from 200℃ to 230℃ at a heating rate of 4℃ min^−1^ and held for 11 min when the temperature reached 230℃. The temperatures of the injector and FID were 250℃ and 260℃, respectively. Hydrogen was used as the carrier gas, the split ratio was set as 10:1, and the injection volume was 1.0 μL. The flow rates of hydrogen and air were 30 ml/min and 300 ml/min, respectively. The determined conditions should meet the theoretical plate number (n) of at least 2000/m and a resolution (R) of at least 1.25. Different individual fatty acids were identified and determined by comparing their retention times and peak areas to those of the standards. The main fatty acids in the membrane were palmitic acid, palmitoleic acid, stearic acid, oleic acid, linoleic acid, and linolenic acid. The content of individual fatty acids was calculated according to the following equation:

$$Xi=\frac{Ai\times m_{\text{Si}}\times F_{\text{TGi}‐\text{FAi}}}{A_{\text{Si}}\times m}\times 100$$

*Xi* represents the content of individual fatty acids in the samples and is expressed in g/kg; *Ai* represents the peak areas of individual fatty acid methyl esters in the tested solution; *m*_Si_ represents the standard quality of a fatty acid triglyceride standard solution; *F*_TGi_ *_‐_*_FAi_ represents the conversion factor of triglycerides to fatty acids; *A*_Si_ represents the peak areas of individual fatty acids in the standard solution; *m* represents the weight of the samples and is expressed in kg;

### 2.6 Activities of energy metabolism‐related enzymes

The activities of succinate dehydrogenase (SDH), cytochrome c oxidase (CCO) H^+^‐ATPase, and Ca^2+^‐ATPase were determined using enzyme‐linked immunosorbent assay (Elisa) kits (Shanghai Jingkang Bioengineering Co., Ltd). The operation was carried out according to the instructions and information provided by the manufacturer. The activities of SDH, CCO, H^+^‐ATPase, and Ca^2+^‐ATPase were expressed as ng/gprot.

### 2.7 Determination of NAD^+^ and NADH contents

The activities of NAD^+^ and NADH were determined using enzyme‐linked immunosorbent assay (Elisa) kits (Shanghai Jingkang Bioengineering Co., Ltd), respectively. The operation was carried out according to the instructions and information provided by the manufacturer. The activities of NAD^+^ and NADH were expressed as ng/gprot.

### 2.8 Activities of membrane metabolism‐related enzymes

The determination of lipoxygenase (LOX) activity, lipase (LPS) activity, and phospholipase (PLD) activity was using lipoxygenase assay kit, lipase assay kit, and phospholipase assay kit (Shanghai Jingkang Bioengineering Co., Ltd), respectively. Their operation was carried out according to the instructions and information provided by the manufacturer. The activities of LOX, LPS, and PLD were expressed as ng/gprot.

### 2.9 Statistical analysis

The experiment was executed under a completely randomized design. All the data from this experiment were calculated using Microsoft Excel 2016 and plotted using Origin Pro 8.5. The data were presented as the means ±standard deviation (*SD*) and their significant difference was analyzed by paired‐sample *t* test in Origin. *p* < .05 was regarded at a significant level according to the test. Correlation coefficients of intracellular indexes and membrane indexes were calculated using Origin Pro 8.5. The correlation was at a significant level when *p* < .05 (Table 1).

**TABLE 1:** Correlation coefficients of intracellular indexes (energy charge, ATP, ATP/ADP, and ATP/AMP) and membrane indexes (lipid peroxidation, membrane permeability, and ratios of unsaturated‐to‐saturated fatty acids) in *Pteridium aquilinum var. latiusculum* during the storage. Correlation coefficients were calculated using Spearman's Correlation in Origin Pro 8.5. *p* < .05 indicated that a significant correlation was observed between the indexes

	Lipids peroxidation (MDA content)			Membrane permeability (RLR)			USFA/SFA		
	*r*	*r^2^*	*p*	*r*	*r^2^*	*p*	*r*	*r^2^*	*p*
USFA/SFA	−0.822	0.758	<.001	−0.842	0.798	<.005	—	—	—
Membrane permeability (RLR)	0.731	0.546	<.05	—	—	—	−0.886	0.746	<.005
Energy charge (EC)	−0.026	0.002	.985	−0.558	0.246	.366	0.052	0.028	.825
ATP	−0.528	0.325	.036	−0.917	0.833	<.001	0.739	0.552	<.05
ATP/ADP	−0.057	0.073	.876	−0.519	0.265	.158	0.625	0.458	<.05
ATP/AMP	−0.034	0.011	.855	−0.436	0.216	.225	−0.044	0.006	.716

## 3 RESULTS AND DISCUSSION

### 3.1 Firmness, TA, weight loss rate, and ethylene production of PA

Firmness is usually regarded as an important index to evaluate the appearance quality of vegetables. The firmness of PA in both the control and treatment decreased gradually during the whole experiment. The decreasing trend of 1‐MCP treatment was obviously delayed compared with that of the control group, and the firmness gap between the two groups tended to be larger as time went on. In contrast, 1‐MCP effectively maintained PA firmness at a relatively high level during storage. This result indicated that 1‐MCP could be an effective preservative to prolong the shelf life of PA during storage.

TA is an important component of vegetable quality and an important factor affecting its flavor quality. The TA content in both the control and treatment groups decreased gradually during storage. Samples treated with 1‐MCP exhibited a slower decrease in TA content than the control. However, the TA content of samples treated with 1‐MCP tended to reach values similar to those of the control by the end of storage. Compared to the control, PA samples treated with 1‐MCP exhibited slower changes in firmness and TA content, which could effectively maintain the appearance quality and edible quality of PA. This result indicated that exogenous 1‐MCP treatment could inhibit the quality changes of PA. Therefore, it could be used for the further investigation of cellular energy status and membrane lipid alterations during PA ripening.

**FIGURE 1:**
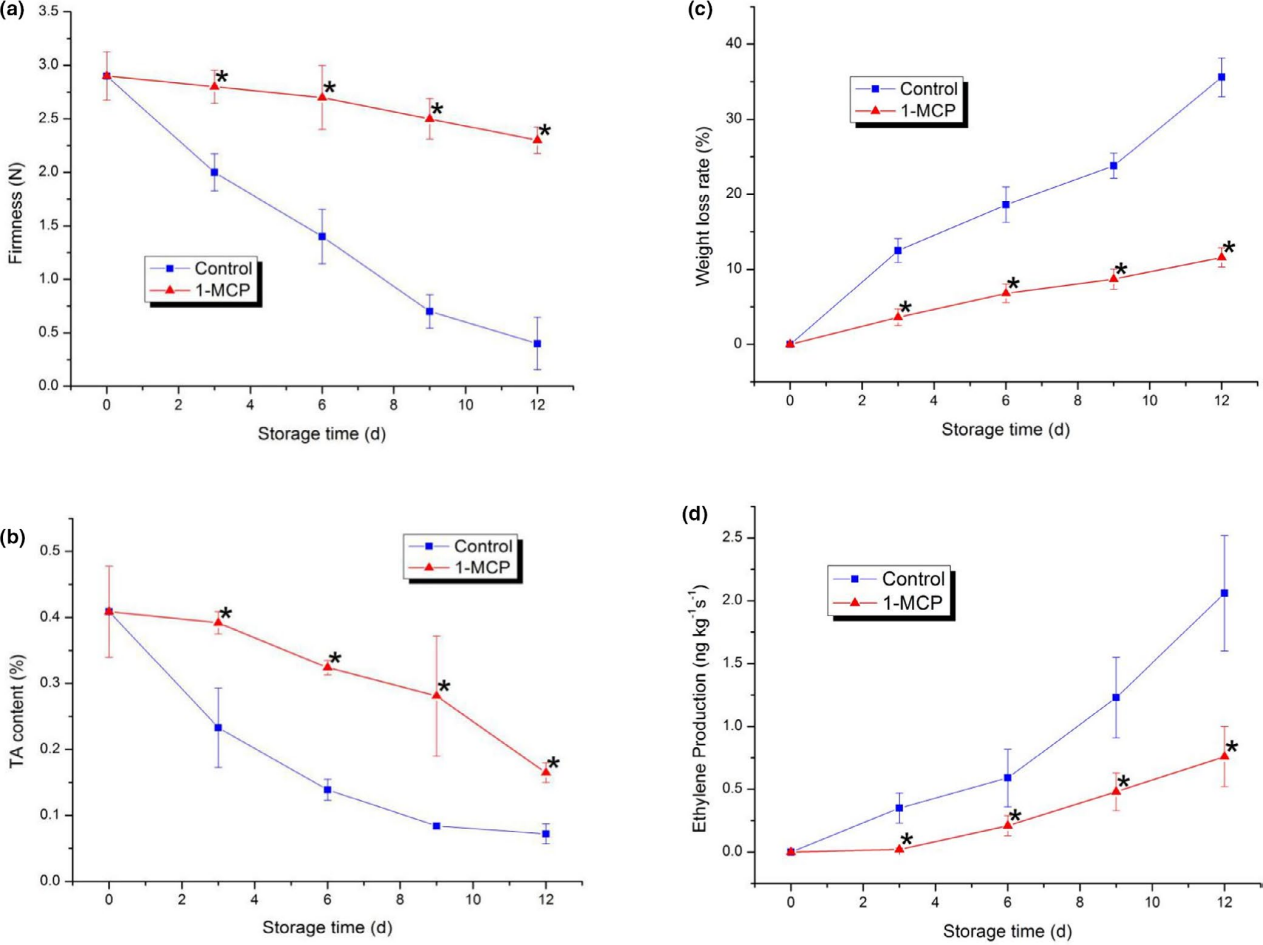
Changes in PA firmness (a), TA content (b), weight loss rate (c), and ethylene production (d) in both the control and the 1‐MCP‐treated sample during postharvest ripening. Each data point is the mean ±standard deviation (*SD*) of three replicates. Asterisks at each time point represent significant differences according to *t* test at *p* < .05

Weight loss rate is an essential index that evaluating the quality of vegetables. Water loss was the main reason for the weight loss in PA. As shown in Figure 1c, the weight loss rates of PA in both groups increased gradually during the storage. The increasing trend of 1‐MCP treatment was significantly lower than that of the control. It indicated that 1‐MCP treatment could inhibit the weight loss of PA and maintain it at a high level effectively.

As shown in Figure 1d, the ethylene production of PA in both groups increased gradually during the whole storage time. However, the endogenous ethylene production of 1‐MCP treated was obviously lower than that of the control during the same storage time. 1‐MCP has been demonstrated to be an effective ethylene inhibitor which could delay the ripening and senescence in the preservation of fruits and vegetables (Rocculi et al., 2009; Salvador et al., 2004; Sharma et al., 2010; Sivakumar & Korsten, 2010). Our data obtained from this study of PA were consistent with some previous reports (Rocculi et al., 2009; Salvador et al., 2004; Sharma et al., 2010; Sivakumar & Korsten, 2010). Consequently, 1‐MCP could inhibit the endogenous ethylene production of PA during the storage time was demonstrated in this study.

### 3.2 Membrane permeability and lipid peroxidation

The relative leakage rate of both the control and treatment increased gradually during storage. Compared to that of the control, the increase in RLR was delayed significantly by 1‐MCP treatment throughout storage. The values of both the control and treatment were similar by the end of the experiment (12 d). This result indicated that the inhibitory effect of 1‐MCP was more effective in the early stage of the experiment.

In general, membrane lipid peroxidation was represented by the accumulation of MDA content. A noticeable increase in MDA was observed in the control during storage, while 1‐MCP treatment significantly delayed the accumulation of MDA. This result indicated that 1‐MCP could effectively inhibit damage to the structure and integrity of the membrane during the ripening process.

The ripening and senescence processes of vegetables are usually accompanied by membrane damage (Ugolini et al., 2017). Membrane permeability and lipid peroxidation are two indexes that evaluate the integrity and stability of plant membranes under biotic and abiotic stresses or during the senescence process (Bajji et al., 2002; Campos et al., 2003; Dhindsa et al., 1981; McCollum & McDonald, 1991). Therefore, 1‐MCP treatment could not only maintain the firmness and TA of PA, but also inhibit the damage to the membrane effectively according to the results of this study.

### 3.3 Energy status

**FIGURE 2:**
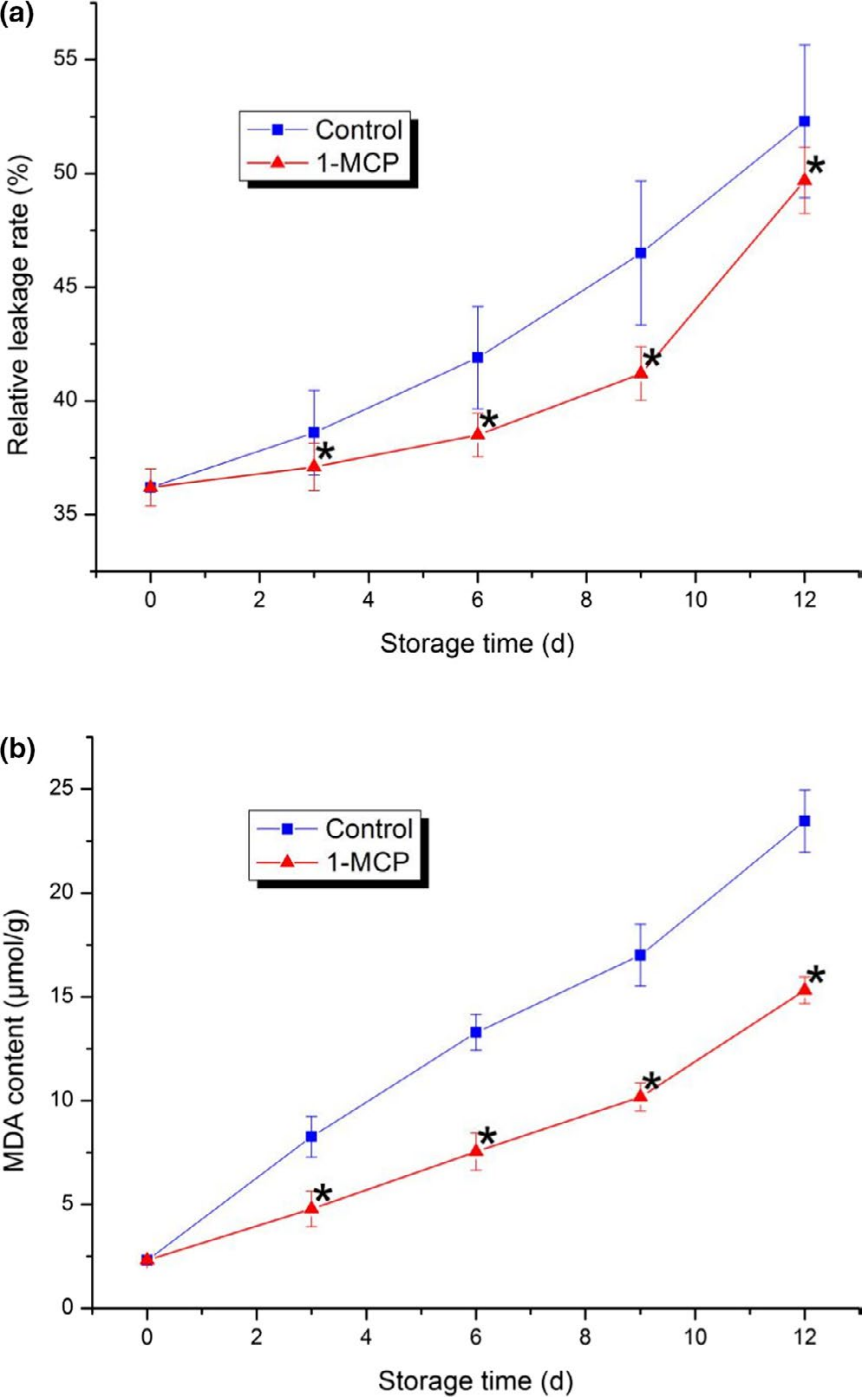
Changes in the PA relative leakage rate (a) and MDA content (b) in both the control and 1‐MCP‐treated sample during postharvest ripening. Each data point is the mean ±standard deviation (*SD*) of three replicates. Asterisks at each time point represent significant differences according to *t* test at *p* < .05

**FIGURE 3:**
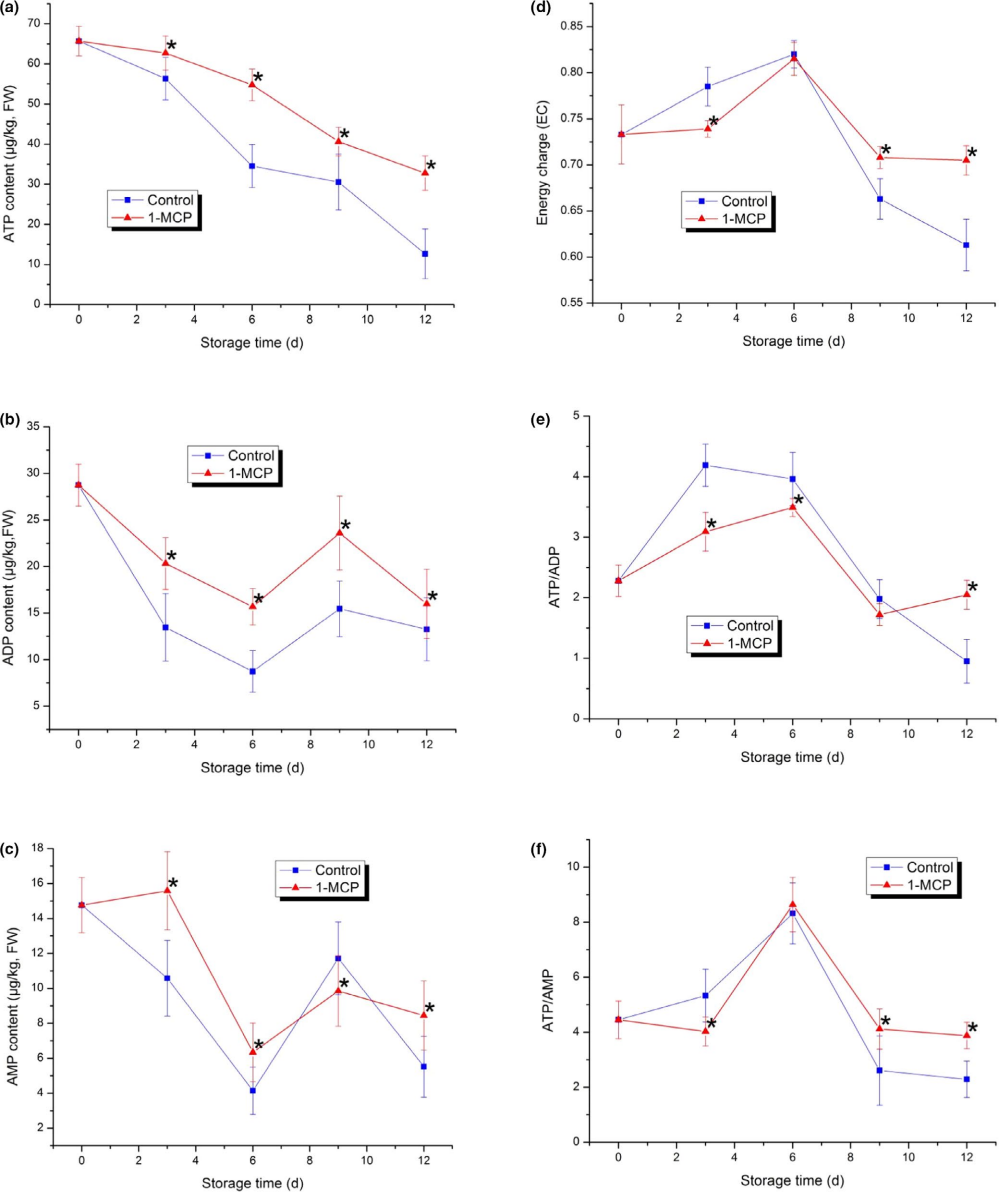
Energy status in PA during the storage. (a) ATP content; (b) ADP content; (c) AMP content; (d) energy status; (e) ratio of ATP/ADP; (f) ratio of ATP/AMP in both the control and 1‐MCP‐treated samples. Each data point is the mean ±standard deviation (*SD*) of three replicates. Asterisks at each time point represent significant differences according to *t* test at *p* < .05

**FIGURE 4:**
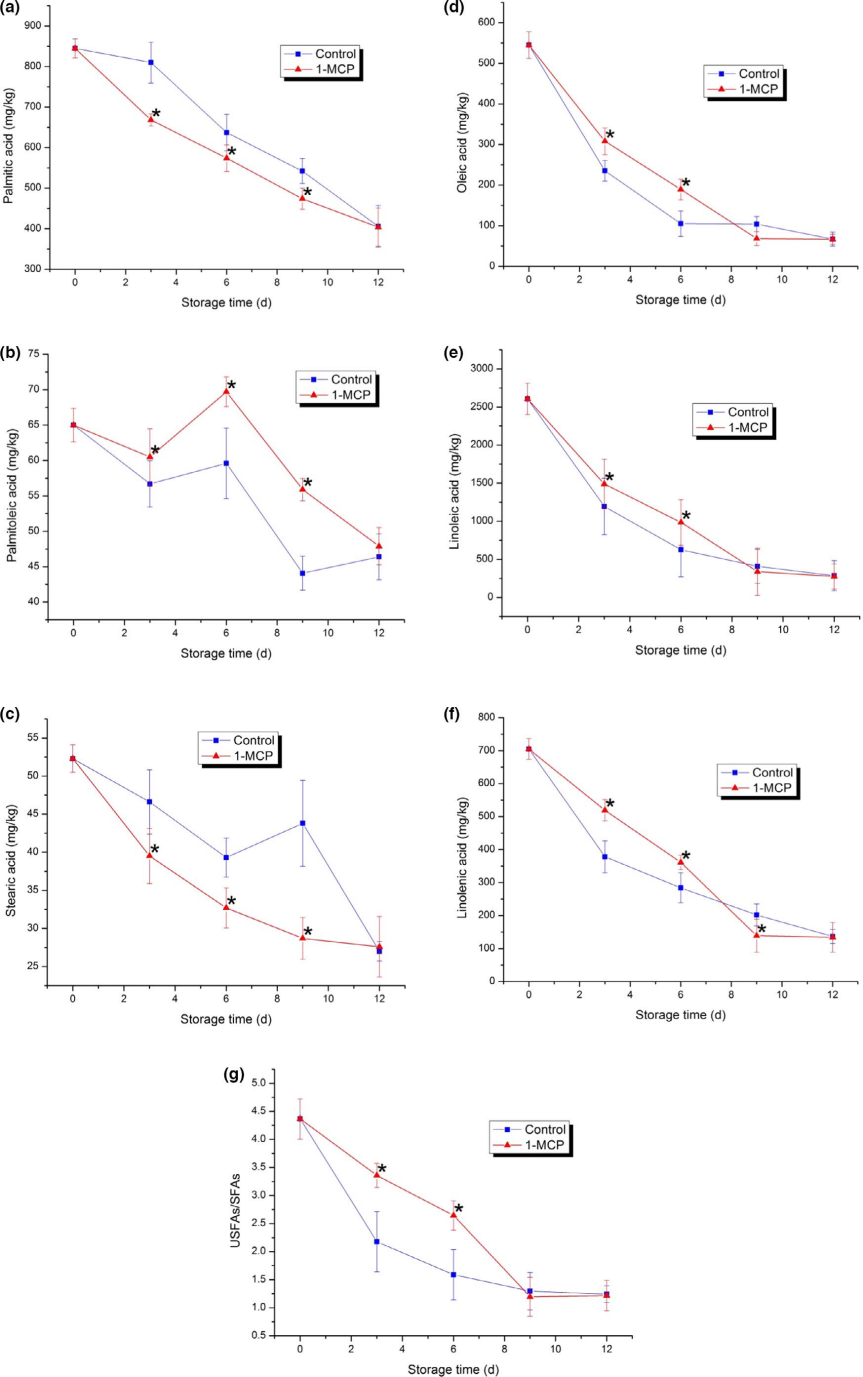
Membrane lipids in PA during storage. (a) Palmitic acid content; (b) Palmitoleic acid content; (c) Stearic acid content; (d) Oleic acid content; (e) Linoleic acid content; (f) Linolenic acid content; (g) Ratio of unsaturated‐to‐saturated fatty acids in both control and 1‐MCP‐treated samples. Each data point is the mean ±standard deviation (*SD*) of three replicates. Asterisks at each time point represent significant differences according to *t* test at *p* < .05

Many previous studies have proven that cellular energy supply is an important factor in controlling ripening and senescence in plants after harvest (Azad et al., 2008; Jiang et al., 2007; Wang et al., 2013). Therefore, inadequate energy supply or a decrease in cellular energy could affect the changes in fruit quality during storage and could even negatively affect physiological disorders and pathogen infection (Huang et al., 2019). In this study, the ATP content showed an obvious downward tendency in both the control and 1‐MCP‐treated sample from the beginning to the end (0–12 d). The ATP content was 65.58 μg/kg initially and decreased to 12.65 μg/kg in the control, 32.77 μg/kg in the 1‐MCP‐treated sample. This indicated that 1‐MCP treatment could delay the decrease in cellular ATP content effectively and maintain it at a relatively high level compared to the control. The ADP content decreased in both the control and 1‐MCP‐treated sample in the first six days. Then, it increased rapidly on the ninth day and decreased to similar levels at 12 d (control: 13.25 μg/kg; 1‐MCP: 15.98 μg/kg). Similar changes were observed in AMP content. The AMP content in the control decreased from 14.76 μg/kg to 4.15 μg/kg in the first six days, then increased to 11.72 μg/kg from the sixth day to the ninth day and decreased rapidly to 5.52 μg/kg by the end of the experiment. The AMP content in the 1‐MCP‐treated sample exhibited a tendency similar to that of the control, while its final content was 8.45 μg/kg, which was slightly higher than that of the control. The decrease in ATP and ADP contents was retarded significantly by 1‐MCP treatment at each detected time point. The changes in ATP content were continuous, while the ADP and AMP contents showed obvious fluctuations in both the control and 1‐MCP‐treated samples during storage. Previous studies have reported that ATP levels are involved in the process of inhibiting pathogen infection and antioxidant property synthesis as well as other exogenous stress responses in some horticulture crops (Aghdam et al., 2018; Li et al., 2014; Yi et al., 2010). Once the senescence process was triggered, the ATP content started to decrease. To maintain the homeostasis of the cellular energy level during storage, the ADP and AMP contents were increased. After increasing for a few days, the ADP and AMP contents also decreased rapidly (Figures 2, 3, 4).

The energy charge was calculated to reveal the overall energy status of the cells in different stages (Jiang et al., 2007; Pradet & Raymond, 1983). ATP/ADP is an important factor related to mitochondrial function by enhancing or inhibiting glycolysis metabolism (Maldonado & Lemasters, 2014). ATP/ADP and ATP/AMP not only are related to senescence, but also affect the incidence of physiological disorders and the alteration of cellular macromolecules (Wang et al., 2003). In this study, the energy charge, ATP/ADP and ATP/AMP all increased in the early storage period and then decreased rapidly in the latter storage period in both the control and 1‐MCP‐treated samples. At the end of the experiment, the energy charge, ATP/ADP and ATP/AMP of the 1‐MCP‐treated samples were all higher than those of the control, and their fluctuations tended to be stable. This indicated that 1‐MCP treatment could effectively inhibit the attenuation of cellular energy status and maintain a stable cellular energy status in PA.

The energy status of harvested horticulture crops has been regarded as one of the major features triggering the senescence process (Aghdam et al., 2018; Saquet et al., 2000; Wang et al., 2013). In this study, the ATP, ADP, and AMP contents; energy charge; and the ATP/ADP and ATP/AMP ratios all decreased at the end of storage, which was consistent with some previous reports (Qu et al., 2006; Saque et al., 2003; Wang et al., 2013). The ATP, ADP, and AMP contents; energy charge; and the ATP/ADP and ATP/AMP ratios in the 1‐MCP‐treated samples were all higher than those of the control at the end of storage. This result indicated that 1‐MCP treatment could retard their decrease effectively. Moreover, prolonged storage time resulted in the accumulation of MDA, an increase in membrane permeability and damage to membrane integrity. These changes were due to the limited availability of both ATP content and energy charge level (Jiang et al., 2007; Saquet et al., 2003). Finally, a significant negative correlation was observed between ATP content and membrane permeability in the harvested PA tissues. Therefore, the senescence process of harvested PA might be closely related to the cellular energy status. Maintaining the ATP level and cellular energy status could retard the quality deterioration of PA.

### 3.4 Changes in membrane fatty acid contents

Changes in membrane fatty acid constituents are considered to be the primary event affecting membrane structure and integrity (Jiang et al., 2007). The composition changes of membrane fatty acids resulting from the decrease in unsaturated fatty acids affected the membrane lipid phase transition (Cao et al., 2011). The detected fatty acid contents decreased over the course of the experiment. The changes in palmitic acid in both the 1‐MCP‐treated sample and the control decreased continuously during storage. This result was opposite to those of some previous reports (Huang et al., 2019; Jin et al., 2014). Stearic acid in the control decreased continuously in the early storage period and increased on the ninth day, and then decreased rapidly. Interestingly, each saturated fatty acid in both the control and 1‐MCP‐treated sample reached a similar level at the end of this experiment. 1‐MCP treatment effectively accelerated the decrease in the saturated fatty acids. Three unsaturated fatty acids (oleic acid, linoleic acid, and linolenic acid) in the 1‐MCP‐treated sample exhibited the same decreasing tendency. In the first six storage days, the contents of three unsaturated fatty acids in the 1‐MCP‐treated sample were higher than those in the control. However, the contents of three unsaturated fatty acids in the control were higher than those in the 1‐MCP‐treated sample on the ninth day, and then they reached the same level at the end of the experiment. The results indicated that 1‐MCP treatment could effectively inhibit the decrease in these three unsaturated fatty acids in early storage. The changes in palmitoleic acid fluctuated obviously but showed a downward trend overall. Palmitoleic acid in the 1‐MCP‐treated sample reached a peak on the sixth day and then decreased rapidly. The unsaturated‐to‐saturated fatty acid ratio (USFA/SFA) of both the control and 1‐MCP‐treated sample was calculated. USFA/SFA in the control decreased continuously during storage and USFA/SFA in the 1‐MCP‐treated sample decreased gradually from 0–9 d and then increased slightly at the end of the experiment (12 d). 1‐MCP treatment obviously inhibited the decrease in the unsaturated‐to‐saturated fatty acid ratio in the early storage period (0–6 d), but the ratio decreased to a level similar to that of the control (6–12 d).

Previous reports revealed that the membrane permeability of apple and potato was related to the saturated fatty acids on the membrane (Knowles & Knowles, 1989; Lurie et al., 1987). The increased unsaturated fatty acids in strawberry could help the plant to adapt to low‐temperature conditions (Wang & Lin, 2006). 1‐MCP combined with heat treatment could inhibit the decrease in linoleic acid and unsaturated‐to‐saturated fatty acid ratios in loquat fruit (Cao et al., 2009). A high USFA/SFA ratio in the membrane could take advantage of preventing the accumulation of MDA and ion leakage, which could avoid peroxidation and damage (Jin et al., 2014). In our study, 1‐MCP treatment retarded the decrease in unsaturated fatty acids and USFA/SFA effectively. This was consistent with the results of some previous studies and indicated that 1‐MCP treatment could maintain membrane integrity and avoid membrane damage effectively. Since the decrease in unsaturated fatty acids might be due to the oxidation of unstable double bonds, whether 1‐MCP treatment affects the fraction of double bonds should be explored in the future.

In our study, USFA/SFA of both the control and 1‐MCP‐treated sample decreased gradually during storage. Significant negative correlations were observed between USFA/SFA and MDA content (lipid peroxidation) and between USFA/SFA and membrane permeability. A significant negative correlation between the unsaturated‐to‐saturated fatty acid ratio and the degree of browning in pear tissues was also observed (Li et al., 2017). These results indicated that the changes in saturated fatty acids, unsaturated fatty acids, and USFA/SFA disrupt the homeostasis of membrane lipids. This might be one of the key factors resulting in damage to membrane integrity in PA during the storage.

Previous studies have demonstrated that the biosynthesis of membrane lipids and the biophysical properties of membranes are affected by cellular energy levels (Jiang et al., 2007). It has also been proven that the synthesis of membrane lipids largely depends on ATP synthesis levels in potato cells (Rawyler et al., 1999). In this study, a negative correlation was observed between cellular energy levels and peroxidation of membrane lipids. Furthermore, oxidation of membrane lipids was regarded to occur due to the unstable double bonds of unsaturated fatty acids (Huang et al., 2019). The homeostasis of membrane lipids was largely maintained by the ratio of unsaturated‐to‐saturated fatty acids. Therefore, the cellular energy status might not be affected by peroxidation of membrane lipids directly. It might be directly affected by the whole status of membrane fatty acids. The ATP level and ATP/ADP were found to be positively correlated with USFA/SFA. Therefore, maintaining or trying to increase the content of unsaturated fatty acids in the membrane could help to protect PA membrane integrity during storage and extend the shelf life of postharvest PA.

### 3.5 Changes in SDH, CCO, H^+^‐ATPase, and Ca^2+^‐ATPase

**FIGURE 5:**
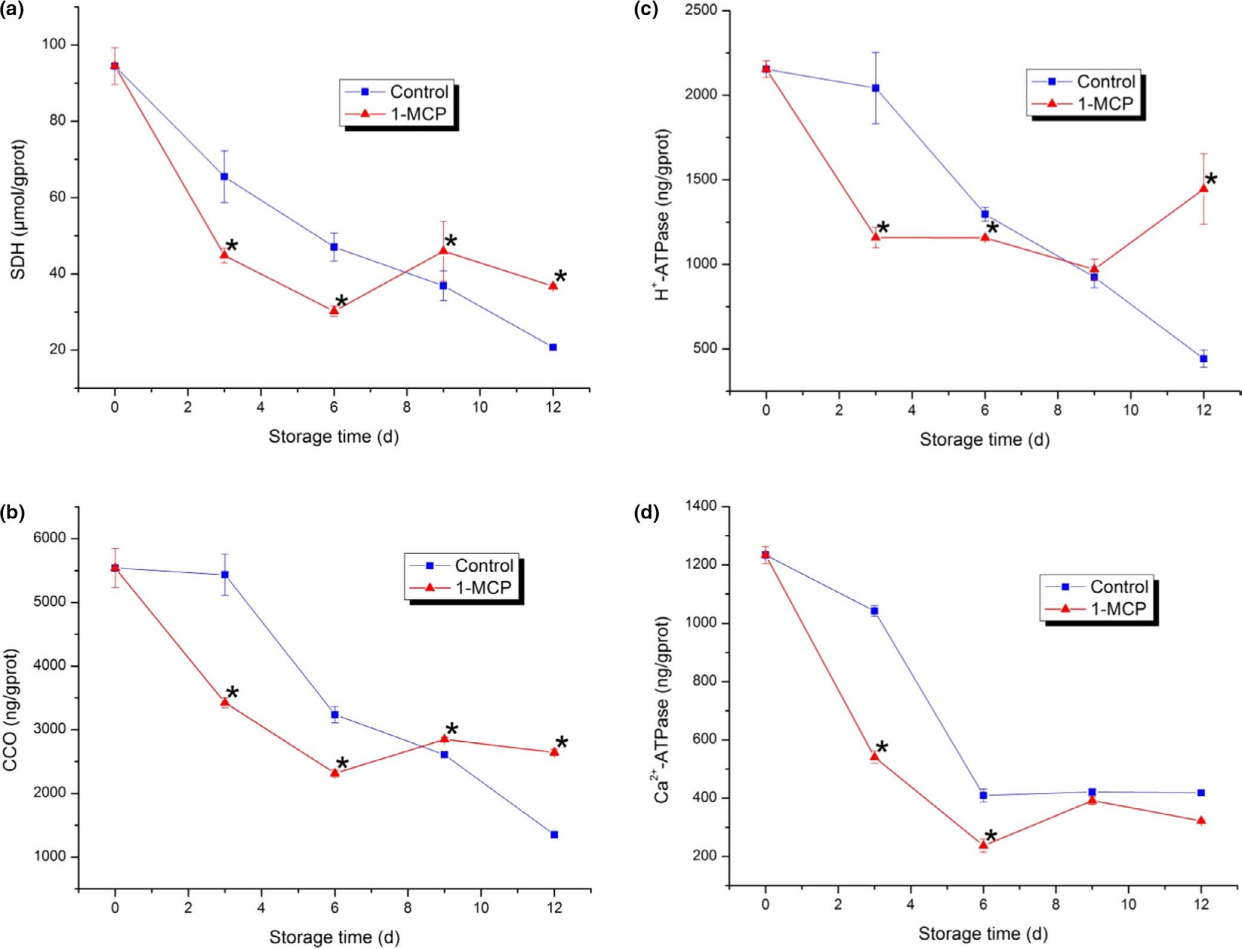
Activities of energy metabolism‐related enzymes in PA. (a) Succinic dehydrogenase (SDH); (b) Cytochrome oxidase (CCO); (c) H^+^‐ATPase; (d) Ca^2+^‐ATPase. Each data point is the mean ±standard deviation (*SD*) of three replicates. Asterisks at each time point represent significant differences according to *t* test at *p* < .05

SDH is a respiration‐related enzyme located on the mitochondrial membranes that participated in electron transport chain (ETC) (Kan et al., 2011). As shown in Figure 5a, SDH activity showed downward tendencies in both the 1‐MCP treatment and the control. In the first six days, SDH activity in the control was higher than that of the 1‐MCP treatment. At the later storage time (9–12 d), SDH activity in the control was significantly lower than that of the 1‐MCP treatment (*p* < .05). This indicated that 1‐MCP treatment could effectively inhibit the decline of SDH activity in PA. CCO is regarded as an electron‐driven proton pump in respiration process (Zhou et al., 2014). For this reason, electron transport will be affected by the changes in CCO activity and then impact on energy metabolism. In this study, CCO activity in control declined persistently and higher than that of 1‐MCP treatment in the first six days. However, CCO activity in 1‐MCP treatment increased slightly and higher than that of the control with significant difference (*p* < .05) at the later storage time (9–12 d). Activities of H^+^‐ATPase and Ca^2+^‐ATPase showed similar decrease trends with those of SDH and CCO. In the first six days, H^+^‐ATPase activity in the control was higher than that of the 1‐MCP treatment. At the end of the storage (12 d), H^+^‐ATPase activity in 1‐MCP treatment was significantly higher than that of the control (*p* < .05). Ca^2+^‐ATPase activity in 1‐MCP treatment was always lower than that of the control during the storage in this study. The difference was significant (*p* < .05) in the first six days while not significant in 9 d–12 d. Ca^2+^‐ATPase activities in both two groups were closed at the end of the storage since Ca^2+^‐ATPase activity in 1‐MCP treatment increased slightly.

ATP is the main cellular energy resource in eukaryotes, and it is mainly generated from oxidative phosphorylation. Oxidative phosphorylation is an important stage of respiration metabolism that occurring in mitochondria (Lin, Chen, et al., 2018; Wu et al., 2016). As an important part of oxidative phosphorylation, ETC consumes oxygen to generate majority of intracellular energy (Møller, 2001). The four enzymes (SDH, CCO, H^+^‐ATPase, and Ca^2+^‐ATPase) involve in producing ATP by playing important roles in ETC process. SDH involves in ETC process via citric acid cycle and provides electrons to the ubiquinone pool (Luo et al., 2020). CCO is the terminal enzyme of ETC in mitochondria which catalyzes the electrons transport from Ferro cytochrome c to molecule oxygen (Soto et al., 2012). Pumping protons across inner mitochondrial membrane generates proton motive force which is utilized by H^+^‐ATPase to produce ATP (Soole & Menz, 2013). The suppression of H^+^‐ATPase activity may result in the decrease of synthesis rate in ATP (Wei et al., 2018). Ca^2+^‐ATPase transports Ca^2+^ from cytoplasm to mitochondria to involve in the subsequent oxidation (Luo et al., 2020). Therefore, stable levels of these four enzymes are required to guarantee energy production.

In our study, the activities of SDH, CCO, H^+^‐ATPase, and Ca^2+^‐ATPase were maintained even enhanced by 1‐MCP treatment. The maintenance of SDH activity might provide a sufficient electron source for ETC. The enhancement of CCO activity might provide a stable electron flow in mitochondria to ensure the generation of proton motive force which was used by H^+^‐ATPase. Although the activity of Ca^2+^‐ATPase was lower than that of the control in the later storage time, 1‐MCP treatment still showed the maintenance in Ca^2+^‐ATPase activity. The results indicated that these enzymes have contributed more production of ATP in 1‐MCP treatment. Some similar results were found in previous studies (Jin et al., 2014; Lin, Lin, et al., 2018; Wei et al., 2018).

### 3.6 NAD^+^ and NADH

The content of NAD^+^ showed significant differences between the 1‐MCP treatment and the control except on day 9. The content of NAD^+^ decreased rapidly in the first three days while increased from day 3 to day 12 persistently in the 1‐MCP treatment and it was three times than that of the control on day 12. The content of NADH in both two groups showed continuous downward trends. The content of NADH in the control was higher than that of the 1‐MCP treatment in the first six days while it was lower than that of the 1‐MCP treatment from day 9 to day 12. The results indicated that 1‐MCP treatment could maintain the content of NADH and enhance the content of NAD^+^ effectively.

**FIGURE 6:**
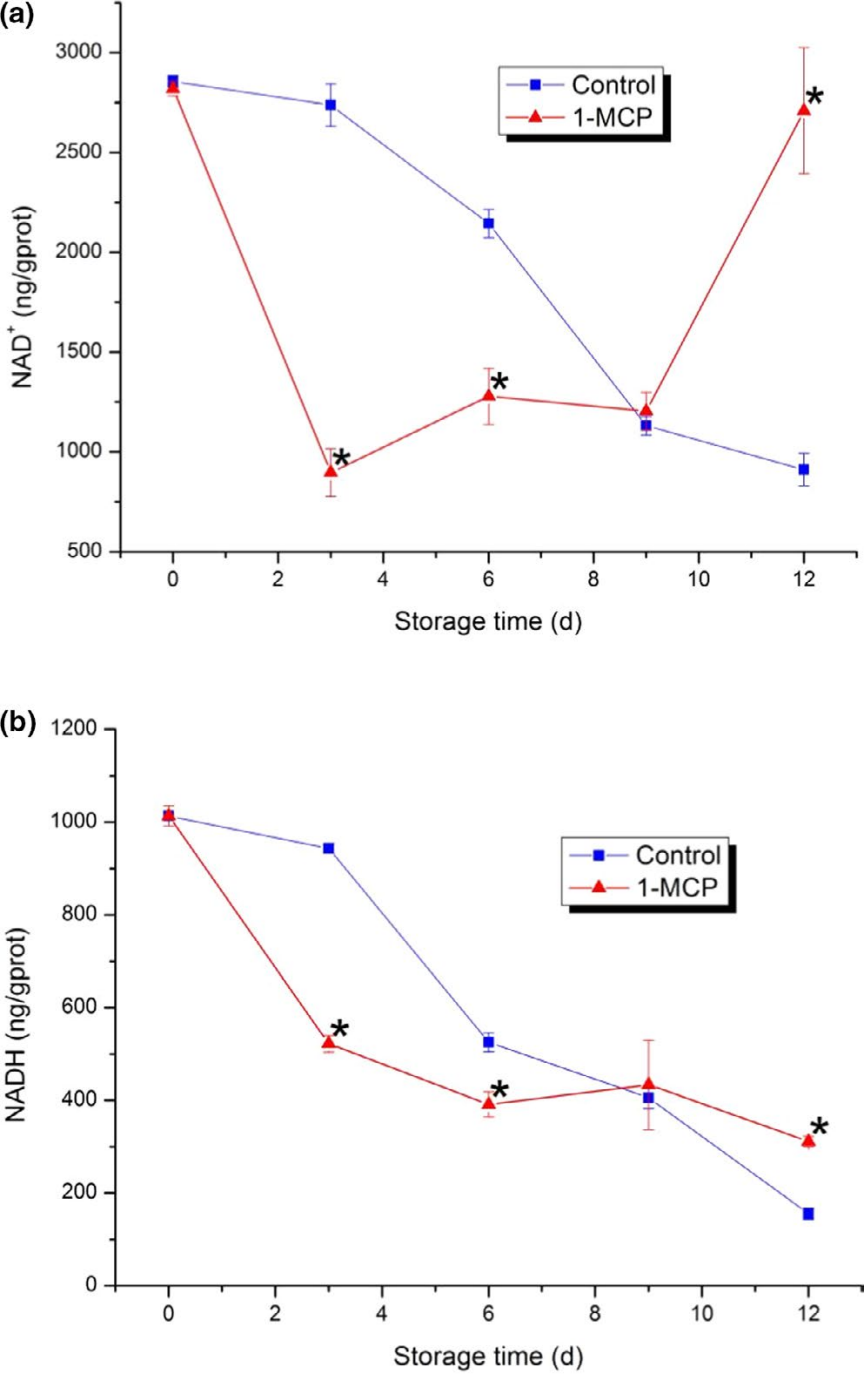
Changes of NAD^+^ and NADH in PA. (a) NAD^+^; (b) NADH. Each data point is the mean ±standard deviation (*SD*) of three replicates. Asterisks at each time point represent significant differences according to *t* test at *p* < .05

NAD^+^ and NADH are redox pairs in cells. As coenzymes of many redox reactions in organisms, they participate in life activities and transform each other (Birkmayer G D, 2009). NADH plays an important role in maintaining cell growth, differentiation, energy metabolism, and cell protection. Stable level of NADH can provide fuel for the ETC process. The amount of NADH is directly related to ATP production. The more NADH each cell contains, the more energy it produces (Chaban et al., 2014). As shown in Figure 6, the content of NAD^+^ and NADH in the 1‐MCP treatment was both significantly higher than those of the control in the later storage time. Since NAD^+^ is oxidized by NADH, the increase of NAD^+^ can prove the energy increase that released by NADH oxidation. In this study, it can be concluded that 1‐MCP treatment can inhibit the decrease of NAD^+^ and NADH effectively, and then to maintain the production of ATP and the level of energy metabolism.

### 3.7 Changes in LOX, LPS, and PLD

**FIGURE 7:**
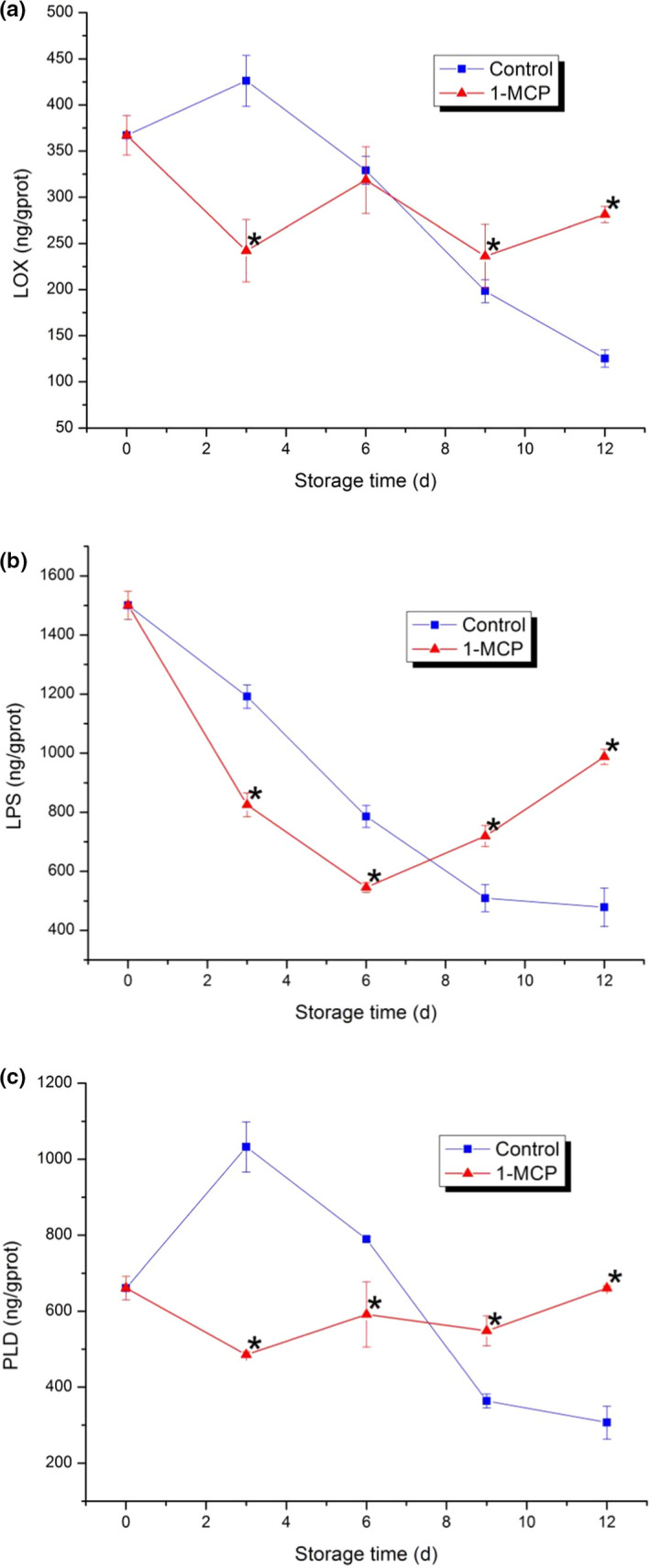
Activities of membrane lipid metabolism‐related enzymes in PA. (a) Lipoxygenase (LOX); (b) Lipase (LPS); (c) phospholipase (PLD). Each data point is the mean ±standard deviation (*SD*) of three replicates. Asterisks at each time point represent significant differences according to *t* test at *p* <.05

**FIGURE 8:**
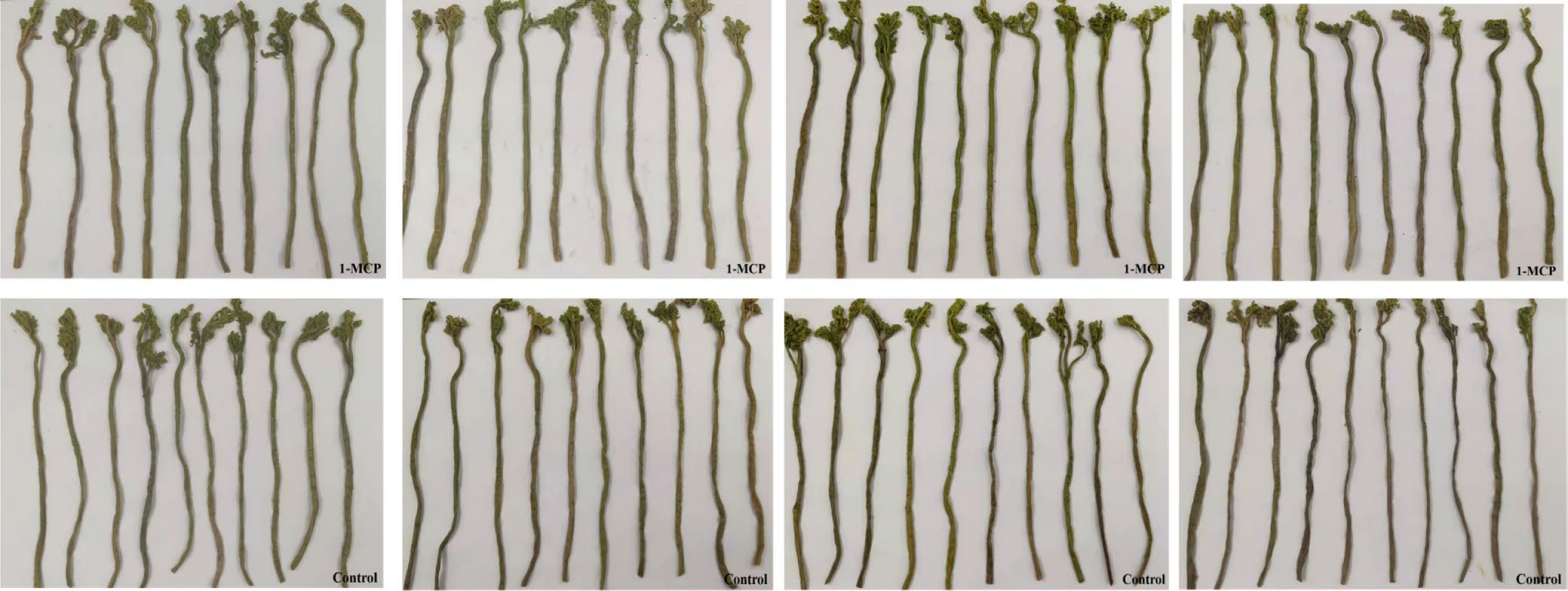
Changes of exterior qualities of the 1‐MCP treatment and the control within 12 days of storage time at 4℃

As shown in Figure 7a, the activity of LOX in the control increased from day 0 to day 3 and decreased gradually from day 3 to day 12. However, the activity of LOX in the 1‐MCP treatment showed obvious fluctuation from day 0 to day 12 and it was higher than that of the control from day 9 to day 12. The activity of LPS in the control decreased persistently during the storage time. The activity of LPS in the 1‐MCP treatment decreased from day 0 to day 6 and then increased from day 6 to day 12. It was higher than that of the control in the end. The changes of PLD activity were similar to the pattern of LOX activity. As shown in Figure 7c, the activity of PLD in the control increased to a peak on day 3 and then decreased persistently from day 3 to day 12. The change of PLD activity in the 1‐MCP treatment showed a steady trend. At the end of the storage, the activity of PLD in the 1‐MCP treatment was higher than that of the control (Figure 8).

Some important enzymes involved in membrane lipids metabolism are associated with the process of membrane lipids peroxidation, such as LOX, LPS, and PLD. In general, LOX can degrade unsaturated fatty acids in membrane. LPS involves in esterifying diacylglycerol to free fatty acids. PLD is regarded as the vital enzyme that initiating the phospholipid catabolism and hydrolyzing the ester bond of phosphoglyceride (Liu et al., 2011). Therefore, the inhibition of these enzyme activities (LOX, LPS and PLD) might explain the role of 1‐MCP in maintaining the normal membrane functions of PA. In this study, the activities of three enzymes were significantly lower than those of the control at the first of the experiment and higher than those of the control at the end of the experiment. The results indicated that 1‐MCP treatment could maintain normal membrane functions via inhibiting the activities of LOX, LPS, and PLD in the early term in PA. It was partially consistent with previous studies (Luo et al., 2020).

In conclusion, 1‐MCP was found to possess the ability to delay senescence in PA, which was indicated by the slower decreases in firmness, TA content and slower increases in weight loss rate, MDA content and membrane permeability, smaller change ranges of energy charge, ATP, ATP/ADP, and ATP/AMP, slower decreases in unsaturated fatty acids and the ratio of USFA/SFA, better maintenance of SDH, CCO, H^+^‐ATPase, and Ca^2+^‐ATPase, higher levels of NAD^+^, NADH, and lower levels of LOX, LPS, and PLD. These results indicated that 1‐MCP treatment could maintain the quality of PA by regulating energy metabolism and membrane lipid metabolism and effectively extend its shelf life.

## CONFLICT OF INTEREST

No conflict of interest exists in the submission of this manuscript, and manuscript is approved by all authors for publication.

## AUTHOR CONTRIBUTION

**Wentao Zhang:** Conceptualization (lead); Formal analysis (lead); Funding acquisition (supporting); Methodology (lead); Validation (lead); Visualization (lead); Writing‐original draft (lead). **Zhen Li:** Data curation (lead); Investigation (lead). **Meiling Du:** Investigation (supporting); Resources (equal). **xiuling zhang:** Funding acquisition (lead); Supervision (lead); Writing‐review & editing (lead). **Yaqin Tian:** Investigation (supporting). **Jinge Wang:** Resources (equal).

## Data Availability

All the data used to support the findings of this study are included within the article.
